# Neuromuscular and Vascular Hamartoma of the Small Bowel: A Rare Cause of Intestinal Obstruction

**DOI:** 10.4103/0974-2727.72213

**Published:** 2010

**Authors:** Vani Krishnamurthy, Vijiya Basavaraj, Sunila, Manjunath Gubbanna Vimalambike

**Affiliations:** JSS Medical College, JSS University, Mysore, India

**Keywords:** Neuromuscular and vascular hamartoma, small intestine, recurrent small bowel obstruction

## Abstract

Neuromuscular and vascular hamartoma (NMVH) is a rare lesion arising chiefly in the small intestine. As it shares many of the histological features with other commonly occurring stricturous conditions of the small bowel, there is an ongoing debate whether it is truly hamartomatous or represents just a reactive condition. We are reporting a case of NMVH in the terminal ileum in a 32-year-old male who presented with symptoms of intestinal obstruction.

## INTRODUCTION

Neuromuscular and vascular hamartoma (NMVH) of the small intestine is an unusual lesion causing intestinal strictures and presenting as recurrent small bowel obstruction or chronic gastrointestinal (GI) bleeding.

It was first described by Fernando and Mc Govern from Sydney in 1982 as a hamartomatous lesion occurring in the small bowel, consisting of fascicles of smooth muscle derived from muscularis mucosa, bundles of unmyelinated nerve fibers with scattered ganglion cells and hemangiomatous vessels occurring focally within a segment of small intestine, causing stenosis.[1]

Since its initial description, only 14 cases have been reported in the English literature.

## CASE REPORT

A male patient, aged 32 years, had recurrent abdominal pain for 2 years, which was being treated as acid peptic disease. He later presented to a General surgeon with abdominal distension and vomiting. He was clinically diagnosed to have intestinal obstruction. A Computerized Tomogram with contrast revealed narrowed terminal ileum with distension of the bowel proximal to it.

Ileo-Ileal intussusception was considered and an exploratory laparotomy was undertaken. A small segment of terminal ileum measuring approximately 7 cm in length was found to be inflamed and narrowed. The proximal segment was dilated. The narrowed segment with adjacent dilated bowel was excised and end to end anastamosis was done. The patient recovered uneventfully after this procedure.

**Figure 1:**
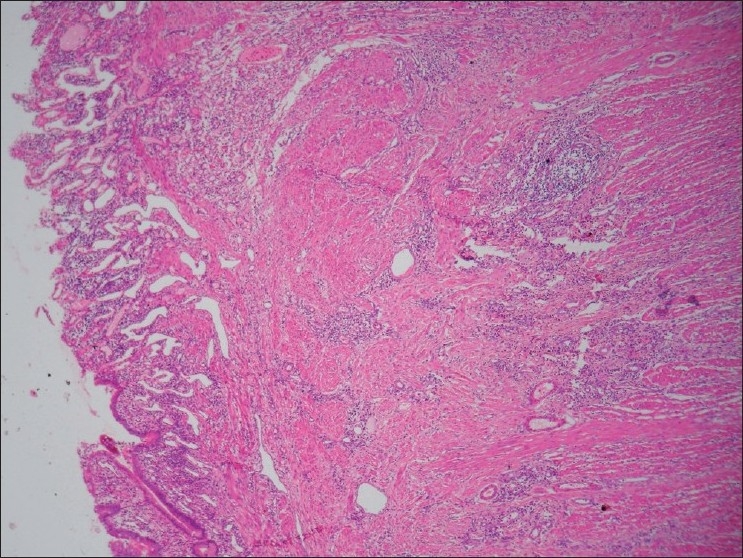
Photomicrograph showing mucosal ulceration, closely packed thin walled vascular channels in the mucosa and expanded submucosa. (H and E, ×40)

The resected ileal segment measuring 12 cm was subjected to histopathology. Proximal portion was dilated and the distal end was narrow. An annular stricture of 5 cm was observed near the distal resected end. Strictured segment showed brown discoloration and irregular superficial ulceration of the mucosa with extensive thickening of the wall. Microscopically, the mucosa showed ulceration, broadening of the villi, extensive crypt hyperplasia, and closely packed, thin-walled vascular channels. Submucosa was markedly expanded by disorganized bundles of smooth muscles in continuity with thickened muscularis mucosa [Figure 1]. Groups of S-100 positive ganglion cells were diffusely scattered in the submucosa along with thickened nerve bundles. Expanded serosa showed similar findings. Lymphoplasmacytic infiltrates of varying density were seen in all the layers.

A diagnosis of NMVH was made.

The procedure proved to be curative and the patient was symptom free at follow-up 25 months later.

## DISCUSSION

NMVH of the small bowel was described by Fernando and McGovern in 1982 as a specific entity. Since then, 14 cases with similar findings have been reported so far.

Macroscopically, majority of the cases showed single or multiple strictures in the jejunum and ileum. Characteristic histological findings included haphazard distribution of fascicles of smooth muscle, ectatic thick-walled vascular channels, thickened unmyelinated nerve bundles and S-100 positive irregularly scattered ganglion cells. Observation of other mesenchymal components suggested an alternative term of neuro mesenchymal hamartoma of the small bowel.[2] Report of a case in the caecum prompted the consideration of NMVH of the small bowel as NMVH of the intestine.[3]

As reported in some studies,[4] similar pathological features may be seen as part of the histological spectrum of Crohn’s disease. Some have observed similar changes also in radiation and ischemic enteritis,[5] and thus Shepherd and Jass[4] concluded that NMVH, in isolation, as a relatively nonspecific change, is probably related to the chronicity of the disease.

Observation of massive submucosal adipose tissue infiltration along with multiple intramural fibrous nodules by Salas *et al*.[2] made them consider that though most of the histological features of NMVH of the small bowel are observed in other common entities, small intramural fibrous nodules are generally an unusual finding. They are not associated with any inflammatory pathology. Hence, they have argued that it is not the nature of the disease nonspecific by themselves, but their intensity and the lack of other more disease-specific alterations that make NMVH a separate entity.

Histological similarity in varied clinical set-up has made the existence of NMVH a debatable issue. The number of cases reported so far is too few to arrive at any conclusion and arguments can be found in each case supporting one or the other opinion. For practical purposes, its unusual appearance fulfills the criteria implicit in the concept of hamartoma, that is, abnormal mixing of normal indigenous tissue components.

NMVH as an entity is controversial. Irrespective of this, there is no doubt that this is an extremely rare lesion. Its features are unique and the prognosis is favorable. It represents a diagnostic challenge for pathologists and a reassuring conclusion for the patient.
